# Exogenous Orexin-A Microinjected Into Central Nucleus of the Amygdala Modulates Feeding and Gastric Motility in Rats

**DOI:** 10.3389/fnins.2020.00274

**Published:** 2020-04-28

**Authors:** Tingting Jin, Zhongxin Jiang, Xiao Luan, Zhuling Qu, Feifei Guo, Shengli Gao, Luo Xu, Xiangrong Sun

**Affiliations:** ^1^Department of Physiology and Pathophysiology, School of Basic Medicine, Qingdao University, Qingdao, China; ^2^Department of Clinical Laboratory, The Affiliated Hospital of Qingdao University, Qingdao, China

**Keywords:** orexin-A, central nucleus of the amygdala, gastric motility, electrophysiology, food intake

## Abstract

Orexin-A is a circulating neuropeptide and neurotransmitter that regulates food intake and gastric motility. The central nucleus of the amygdala (CeA), which regulates feeding behavior and gastric function, expresses the orexin-1 receptor. The aim of this study was to evaluate the effects of microinjection of exogenous orexin-A into the CeA, on food intake and gastric motility, and to explore the mechanisms of these effects. Normal chow and high fat food (HFF) intake were measured, gastric motility and gastric emptying were evaluated, extracellular single unit firing was recorded, and c-fos expression was determined. The results showed that microinjection of orexin-A into the CeA resulted in increased HFF intake but did not affect normal chow intake. This effect was blocked by an orexin-1 receptor antagonist-SB-334867 and was partially blocked by a dopamine D1 receptor antagonist-SCH-23390. Gastric motility and gastric emptying were enhanced by orexin-A, and the former effect was abolished by subdiaphragmatic vagotomy. The firing frequency of gastric distention-related neurons was regulated by orexin-A *via* the orexin-1 receptor. Furthermore, c-fos expression was increased in the ventral tegmental area (VTA) and the nucleus accumbens (NAc), the lateral hypothalamus (LHA), and the dorsal motor nucleus of the vagus (DMV) in response to microinjection of orexin-A into the CeA. These findings showed that orexin-A regulated palatable food intake and gastric motility *via* the CeA. The LHA, the VTA, and the NAc may participate in palatable food intake and the CeA-DMV-vagus-stomach pathway may be involved in regulating gastric motility through the regulation of neuronal activity in the CeA.

## Introduction

Food intake is regulated by several interrelated brain regions, including the hypothalamus, the hindbrain, and the limbic system (Merali et al., 2013; Abdalla, 2017; Davis and Grill, 2018). The amygdala is an important limbic structure involved in a range of motivationally and emotionally driven behaviors such as fear, anxiety, reward, and memory. Recent studies showed that the amygdala can integrate feeding-related signals from the cortex, the hypothalamus, and the hindbrain to regulate food intake (Sweeney and Yang, 2017; Ishida et al., 2018; Hardaway et al., 2019). However, the mechanisms that underlie the role of the amygdala in feeding have not been elucidated. The amygdala also plays an important role in modulation of gastrointestinal motility and secretion (Feng et al., 2007; Wang et al., 2014; He and Ai, 2016). Mood change, whether positive or negative, is associated with alterations in feeding and gastric functions (Huerta-Franco et al., 2012; Porcelli et al., 2014; Abizaid, 2019; Cassidy et al., 2019; Israelyan et al., 2019). However, the role of the emotional center in the digestive system is largely unknown. The amygdala is comprised of the basolateral complex of the amygdala (BLA), the central nucleus of the amygdala (CeA), and the corticomedial nucleus of the amygdala. The CeA, similar to the hypothalamus, plays an essential role in feeding behavior in both rodents and humans (Areias and Prada, 2015; Kim et al., 2017). Although projections from the CeA may reach the feeding-related centers in the hypothalamus and/or the hindbrain, the exact neural pathways that control feeding behavior require further study. In addition, anatomical and physiological studies have shown that connections between the CeA and the dorsal vagal complex (DVC), which is a regulatory center for visceral function, affect gastric motility (Liubashina et al., 2000; Zhang et al., 2003; Cassidy et al., 2019).

Orexin is a circulating neuropeptide and neurotransmitter originally isolated from the medial perifornical region (PeF), the dorsomedial hypothalamus (DMH), and the lateral hypothalamus (LH) (de Lecea et al., 1998; Sakurai et al., 1998). Orexin-A (hypocretin-1) and orexin-B (hypocretin-2) are the two major isoforms of orexin, and these isoforms interact with the orexin-1 receptor (OX1R) and the orexin-2 receptor (OX2R). Studies have shown that OX1R has higher affinity for orexin-A, and OX2R has equal affinity for orexin-A and orexin-B (Sakurai et al., 1998). Human and animal studies have shown that orexin-A is important in the control of food intake, body weight, and energy expenditure (Lubkin and Stricker-Krongrad, 1998; Yamanaka et al., 1999; Perez-Leighton et al., 2012). Orexinergic neurons send dense projections to many brain regions, including the limbic system (Peyron et al., 1998; Schmitt et al., 2012). Orexinergic fibers from the PeF have been shown to regulate neuronal activity in the CeA through OX1R, and to evoke behavioral responses associated with fear-related disorders (Dustrude et al., 2018). Pharmacological studies showed that OX1R is the main receptor subtype in the CeA and the ventral tegmental area (VTA) involved in binge-like EtOH drinking (Olney et al., 2017). However, the role of OX1R in the CeA in regulation of feeding and gastric function has not been characterized. In this study, we investigated the effects of microinjection of exogenous orexin-A into the CeA on food intake, gastric emptying, and gastric motility. We also explored possible mechanisms of these effects.

## Materials and Methods

### Animals

Male Wistar rats (250–300 g) were housed in an environment with relative humidity 60 ± 5%, temperature 23 ± 2°C, and light from 7:00 am to 7:00 pm. Chow pellets or palatable high fat pellets and tap water were available *ad libitum*. The rats were provided by the Qingdao Institute of Drug Control. The experiments were performed according to the National Institutes of Health Guide for the Care and Use of Laboratory Animals (NIH Publications No. 8023) and its 1978 revision. All procedures in the experiment were approved by the Institutional Animal Care and Use Committee of Qingdao University.

### Brain Surgery

The rats were anesthetized *via* an intraperitoneal injection of thiobutabarbital (100 mg/kg, i.p) and placed in a stereotactic apparatus (Narashige SN-3, Tokyo, Japan). A cannula (stainless steel, 24-gauge) for drug injection was implanted into the CeA [bregma: P: 2.5 mm, L(R): 4.2 mm, H: 8.0 mm] (Paxinos and Watson, 2007) on both sides and fixed to the skull using stainless steel screws and dental acrylic. A stainless-steel stylet was used to seal the cannula to prevent blockage. Following cannulation, the rats were administered intraperitoneal injections of penicillin for three days. The rats were allowed to recover for at least 1 week prior to use in experiments.

### Measurement of Food Intake

Forty-two rats were cannulated and randomly assigned to the following seven groups (*n* = 6 in each group): NS; 0.05 μg orexin-A; 0.5 μg orexin-A; 5 μg orexin-A; 5.0 μg SB-334867 (OX1R antagonist); 0.5 μg orexin-A + 5.0 μg SB-334867; and 0.5 μg orexin-A + 2.0 μg SCH-23390 (dopamine D1 receptor antagonist). The injection volume for each group was 0.5 μl. The rats were fasted for 18 h, then injected at 9:00 am on the following morning with drugs, into the CeA through the cannula using a needle connected to a syringe by a polyethylene tube. Then, pre-weighed chow diet or palatable HFF [50% fat (82% lard and 18% vegetable oil), 25% carbohydrate (30% dextrin, 30% cornstarch and 40% sucrose), and 25% protein (100% casein)] (Rada et al., 2012) was placed in the home cages with the rats. The remaining food was weighed separately at 2 and 4 h after administration, and the remaining weight was subtracted from the initial weight to determine food intake.

### Gastric Emptying

The gastric emptying rate was determined by injecting a phenol red solution into the stomach, as previously described (Sun et al., 2017). Thirty-six rats underwent cannulation and were randomly assigned to the following six groups (*n* = 6 in each group): NS; 0.05 μg orexin-A; 0.5 μg orexin-A; 5 μg orexin-A; 5.0 μg SB-334867; 0.5 μg orexin-A + 5.0 μg SB-334867. The injection volume was 0.5 μl. The drug was injected into the CeA through the cannula. Ten minutes after drug administration, 1.5% carboxymethylcellulose sodium salt containing 0.05% phenol red (1.5 ml/rat) was introduced into the stomach by gavage. The rats were sacrificed 20 min after gavage. The stomach contents were measured using a spectrophotometer to determine residual phenol red. Gastric emptying was determined using the following equation: Gastric emptying rate (%) = (1– amount of test sample/amount of standard sample) × 100%

### Measurement of Gastric Motility

Thirty-six rats with implanted cannulas were randomly assigned to the following six groups: NS; 0.05 μg orexin-A; 0.5 μg orexin-A; 5 μg orexin-A; 5 μg SB-334867; 5 μg SB-334867 + 0.5 μg orexin-A. The injection volume was 0.5 μl. After 18 h of fasting, the rats were anesthetized with 10% chloral hydrate (0.3 mL/100 g). Then, the rats were fixed in the supine position and abdominal surgery was performed to expose the stomach. The stress sensor was sewn 0.3-cm upward from the stomach pylorus into the outer layer of the gastric antrum along the axis of the antral ring of the gastric antrum. The wire of the stress sensor was wound subcutaneously from the back of the rat to the posterior neck, then fixed through the posterior neck skin incision. A 2–3-cm wire was placed on the body surface for connection to the recorder, and the abdomen was closed. Then, 80,000 units of penicillin was intraperitoneally injected for 3 days to prevent infection.

After recovery, the rats were fasted overnight, then placed in the experimental cage for 1 h to adapt to the environment. Gastric motility was recorded using a Polygraph (3066-23; Chengdu Precision Instruments, Sichuan, China) by connecting the transducer lead wires to a bridge circuit. After recording baseline motility for 20 min, the drug was injected into the CeA through the cannula. Recordings were collected for 1–2 h per day for several days with at least a 2-day interval between recordings. The effects of the treatments on gastric motility were evaluated by change in motor index (%MI) per 5 min. Change in motor index was calculated as follows: 100% × (area under the manometric trace after drug injection)/(area under the manometric trace before drugs injection).

### Vagus Nerve Resection

Twenty-four cannulated rats were assigned to the following four groups (*n* = 6 in each group): sham operation + NS, sham operation + 0.5 μg orexin-A, vagotomy + NS, vagotomy + 0.5 μg orexin-A. The injection volume was 0.5 μl. The rats were fasted for 18 h prior to the experiment. The rats were anesthetized by intraperitoneal injection of barbital (100–150 mg/kg). After anesthetization, the rats were fixed in the supine position. The abdominal skin was cut along the abdominal white line and the esophagus and stomach were exposed. The subphrenic vagus nerve was separated around the esophagus near the gastric cardia, and the ventral and dorsal branches were cut. The sham operation group only underwent separation of the subphrenic vagus nerve. Following this procedure, the abdomen was closed. Penicillin was given postoperatively for three consecutive days to prevent infection. The rats were allowed to recover for at least 3 days prior to evaluation of gastric motility. Changes in gastric motility were evaluated by the change of gastric amplitude and gastric frequency before and after drug injection.

### Electrophysiology

Polyethylene tubing (PE-240) with a latex balloon was surgically inserted into the stomach to cause distension. The balloon was inflated using warm saline (37°C, 3–5 ml) at a rate of 0.5 ml/s, and distension was maintained for approximately 10–30 s.

A four-tube glass microelectrode (electrode tip diameter 5–10 μm, electrode impedance 5–15 Ω) was implanted into the CeA [bregma: P: 2.3–2.8 mm, L(R): 4.1–4.3 mm, H: 7.7–8.2 mm]. One tube was filled with 0.5 M sodium acetate and 2% pontamine sky blue for use as the recording electrode. The other three tubes were filled with orexin-A (15 nM), SB-334867 (25 nM), or physiological saline. Each treatment solution was sprayed onto the surface of the cells using short-pulse gas pressure (1500 ms, 5.0–15.0 psi). When the microelectrode reached the border of the air and the agar surface, the hydraulic thruster was used to advance the microelectrode into the CeA. The discharge signal was amplified using an MEZ8201 amplifier (Nihon Kohden, Tokyo, Japan) and displayed on an oscilloscope (VC-II, Nihon Kohden, Tokyo, Japan). All data were stored in a computer and analyzed using the SUMP-PC bioelectric signal processing system. After firing had stabilized for at least 20 min, the average frequency 120 s prior to drug administration was calculated as the basal frequency. Then, the neuron underwent GD stimulation to determine if it received input signals from the gastric mechanoreceptor. Neurons were identified as GD-responsive neurons if the average firing frequency of the neurons changed by at least 20% from the average basal firing level in response to gastric expansion. Gastric distension-responsive neurons were further divided into GD-excitatory (GD-E) neurons and GD-inhibitory (GD-I) neurons according to their responses to GD.

### Immunofluorescence Staining

Rats were randomly assigned to the following two groups: orexin-A (*n* = 8) and saline (*n* = 8). Two hours after administration of 0.5 μg of orexin-A or saline through the cannulas into the CeA, the rats were fixed by perfusion with 0.9% saline and 4% paraformaldehyde. After dehydration using 30% sucrose, the brains were coronally sectioned into 15-μm slices. A series of coronal sections were obtained from the nucleus accumbens (NAc), the VTA, the lateral hypothalamic area (LHA), the arcuate nucleus (ARC), the nucleus of the solitary tract (NTS), and the dorsal motor nucleus of the vagus (DMV), according to a rat brain atlas (Paxinos and Watson, 2007). The sections were incubated with mouse anti-c-fos antibody (1:800, Santa Cruz Biotechnology, United States) overnight at 4°C. On the next day, the sections were incubated with Cy3-binding goat anti-mouse antibody (1:300, Jackson ImmunoResearch, West Grove, PA, United States) for 2 h at room temperature. Fluorescence was visualized using a BX63F fluorescence microscope, and images were recorded using a DP80 digital camera (Olympus, Tokyo, Japan).

To count the number of c-fos immunopositive neurons, six discontinuous sections from each tested brain area were selected according to the size of the nuclei, and 4–6 non-overlapping 0.01-mm^2^ fields were selected from each section. Neurons were defined as c-fos-positive when nuclear staining was evident, and the nuclear border was distinct from the background. CellProfiler-3.1.8 was used to count cells, and the results were expressed as the number of cells per 0.01 mm^2^.

### Histological Verification

**FIGURE 1:**
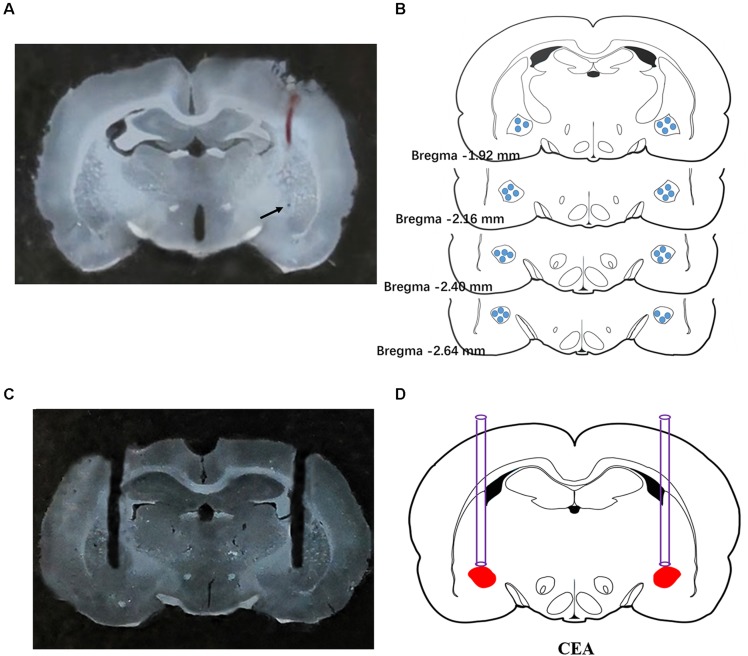
Histological verification of the extracellular recording and microinjection sites. A typical photomicrograph indicated the discharge recording site in CeA **(A)**. Arrow showed the injected position of pontamine sky blue. Brain structure diagrams of coronal sections revealing the single unit recording sites **(B)** and a typical photomicrograph revealing the site of microinjection was confined to CeA **(C)**. Microinjection position was shown in the rat brain atlas **(D)**.

To verify the extracellular recording site, pontamine sky blue was ejected from the microelectrode to the recording site by iontophoresis (10 μA, 20 min) at the end of the experiment. The rats were perfused with physiological saline and paraformaldehyde, and 50-μm-thick frozen coronal sections were prepared following removal of the brain. The tissue sections were visualized using a light microscope. Sections with pontamine blue sky staining that was not centered in the CeA were excluded from the analysis. Fifty rats were subjected to electrophysiological experiments, and 153 neurons were recorded. Sixteen neurons (10%) were not analyzed due to misalignment of the microelectrodes. A typical photomicrograph indicated discharge recording site in CeA was shown in Figures 1A,B.

To verify the position of implanted cannulas, the traces of the cannulas were visualized in 100-μm-thick frozen brain coronal sections (Figures 1C,D). A total of 166 rats were implanted with cannulas, and 12 rats (7.2%) were excluded from data analysis due to improperly located cannulas.

### Drugs and Statistical Analysis

Orexin-A and SB-334867 were purchased from Tocris (Bristol, United Kingdom). SCH-22390 was purchased from Sigma-Aldrich (St Louis, MO, United States) Mouse anti-c-fos antibody was purchased from Santa Cruz Biotechnology (Santa Cruz, CA, United States) and Cy3-binding goat anti-mouse antibody purchased from Jackson ImmunoResearch (West Grove, PA, United States). The data were analyzed using Prism 6 (GraphPad Software, San Diego, CA, United States), and the results were expressed as the mean ± SD. Paired Student’s *t*-test was used to analyze changes in discharge rate before and after treatment. Student’s *t*-test was used to compare differences between two groups, and one-way or two-way analysis of variance followed by Bonferroni *post hoc* test were used to compare differences among three or more groups. *P* < 0.05 was considered statistically significant.

## Results

### Effect of Microinjection of Orexin-A Into the CeA on Food Intake

**FIGURE 2:**
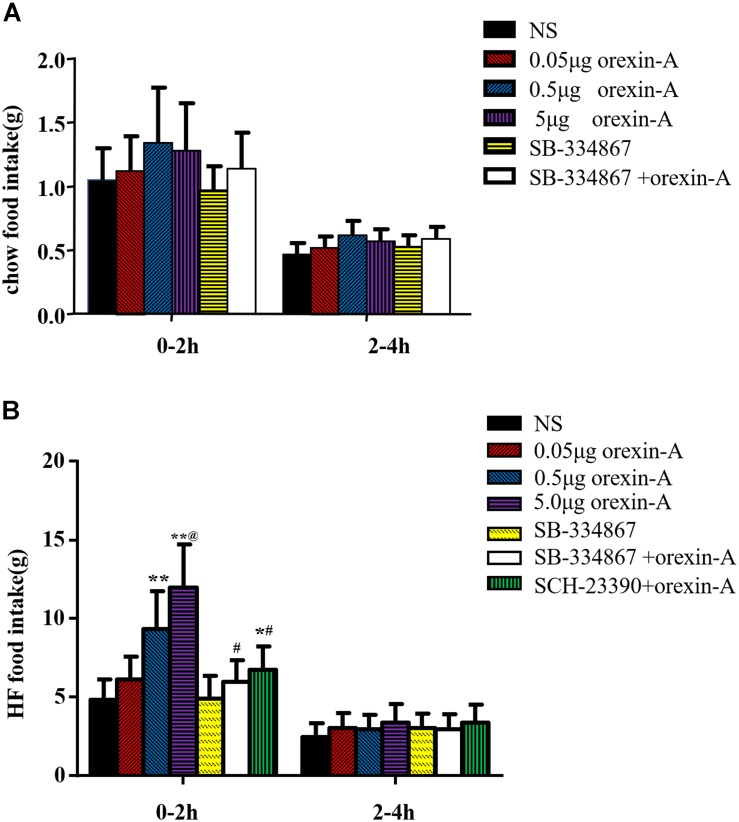
The effect of Orexin-A on food intake in rats. After injection of orexin-A into CeA, the chow food intake of the rats did not change during the 0–2 h **(A)** period and 2–4 h **(A)**. The high fat food intake of the rats increased during the 0–2 h **(B)** period after micro-injection of orexin-A, but this phenomenon did not appear in 2–4 h **(B)**. SB-334867 completely and SCH-23390 partially blocked the action of orexin-A. Data are represented as mean ± SD, *n* = 6. Data were analyzed using one-way ANOVA followed by a Bonferroni *post hoc* test to compare the effect of different orexin-A groups and student’s *t*-test was used to determine the effect of SB-334867 and SCH-23390. *p < 0.05, **p < 0.01 vs. NS group; ^#^*p* < 0.05 vs. 0.5 μg orexin-A group. ^@^*p* < 0.01 vs. 0.05 μg orexin-A group. NS, normal saline.

Injection of 0.5 μl of orexin-A (0.05, 0.5, 5 μg) into the CeA did not significantly affect chow food intake at 0–2 h or 2–4 h compared with that in the NS group. Furthermore, 5.0 μg of SB-334867 alone did not affect chow food intake (*P* > 0.05, Figure 2A).

At 0–2 h, orexin-A increased palatable HFF intake in a dose-dependent manner. Furthermore, 0.5 μg orexin-A and 5 μg orexin-A induced significantly higher HFF intake than that in the NS group [*F*(3,20) = 14.55, *P* < 0.01, Figure 2B]. However, these differences were not observed at 2–4 h (*P* > 0.05, Figure 2B). In addition, administration of 5.0 μg of the OX1R antagonist SB-334867 completely reversed the 0.5 μg orexin-A-induced increase in HFF intake (*P* > 0.05, compared with NS group; *P* < 0.05, compared with the 0.5 μg orexin-A group, Figure 2B). Furthermore, treatment with 2.0 μg of the dopamine D1 receptor antagonist SCH-23390 partially reversed orexin-A induced increases in high fat food intake (*P* < 0.05, compared with the NS group; *P* < 0.05, compared with the 0.5 μg orexin-A group, Figure 2B). Administration of SB-334867 alone did not affect high fat food intake.

### Effect of Orexin-A in the CeA on Gastric Emptying

**FIGURE 3:**
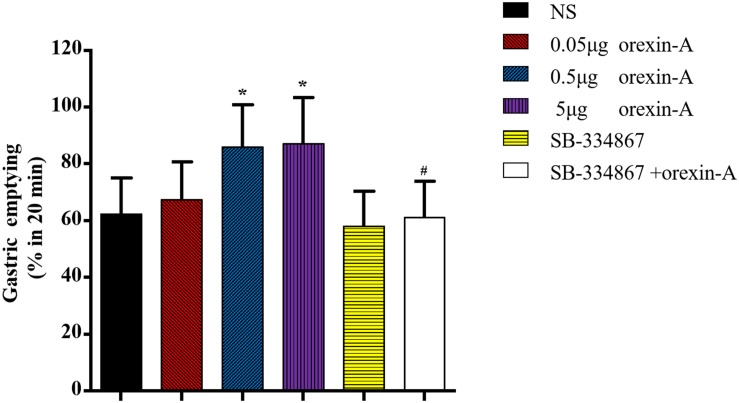
Effects of orexin-A in the CeA on the gastric emptying. 0.05, 0.5, 5 μg orexin-A, 5.0 μg SB-334867 or 0.5 μg orexin-A + 5.0 μg SB-334867 was microinjected into CeA from cannula on the head of rats, respectively. 0.5 and 5 μg orexin-A increased gastric emptying obviously and the effect of 0.5 μg orexin-A could be blocked by 5.0 μg SB-334867. Data are represented as mean ± SD, *n* = 6. Data were analyzed using one-way ANOVA followed by a Bonferroni *post hoc* test to compare the effect of different orexin-A groups and Student’s *t*-test was used to determine the effect of SB-334867. *P < 0.05, compared with NS group; ^#^*P* < 0.05, compared with 0.5 μg orexin-A group. NS, normal saline.

To further investigate the role of the CeA in orexin-A-induced changes in gastric function, we microinjected a range of doses of orexin-A into the CeA and evaluated gastric emptying. The 20-min gastric emptying rate was 86.04 ± 14.78% in response to 0.5 μg of orexin-A and 87.27 ± 16.11% in response to 5 μg of orexin-A. These rates were significantly different from that in the control group [NS: 62.34 ± 12.83%; *F*(3,20) = 4.761, *P* < 0.05, Figure 3]. Treatment with 5 μg of SB-334867 inhibited 0.5 μg orexin-A-induced changes in gastric emptying (61.24 ± 12.75%, *P* < 0.05 compared with the 0.5 μg orexin-A group, Figure 3). These results indicated that orexin-A enhanced gastric emptying via signaling through OX1R.

### Effect of Microinjection of Orexin-A Into the CeA on Contraction of the Gastric Antrum

**FIGURE 4:**
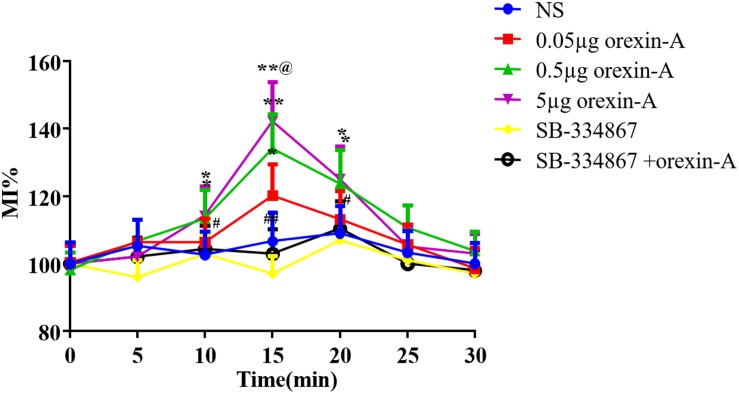
Effect of CeA micro-injection of orexin-A on gastric smooth muscle contraction. 0.05, 0.5, 5 μg orexin-A, 5 μg SB-334867 or 0.5 μg orexin-A + 5 μg SB-334867 was microinjected into CeA from cannula on the head of rats, respectively. The changes of gastric smooth muscle contraction were evaluated by the percentage motor index (%MI) of the motor activity in the antrum in every 5 min. %MI = 100% × (area under the manometric trace after drugs injection)/(area under the manometric trace before drugs injection). Data are represented as mean ± SD, *n* = 6. Data were analyzed using one-way ANOVA followed by a Bonferroni *post hoc* test to compare the effect of different orexin-A groups and Student’s *t*-test was used to determine the effect of SB-334867. *P < 0.05, **P < 0.01 compared with NS group; ^#^*P* < 0.05, ^##^*P* < 0.01, compared with 0.5 μg orexin-A group. ^@^*p* < 0.01 vs. 0.05 μg orexin-A group. NS, normal saline.

To characterize the mechanism of orexin-A-induced acceleration of gastric emptying, we evaluated the effect of orexin-A on contraction of the smooth muscle of the antrum in freely moving rats. Following injection of orexin-A into the CeA, we measured the percent motor index of gastric motility (%MI) every 5 min for 30 min. Changes in %MI were apparent from 10 to 20 min following administration of orexin-A, and the peak change occurred at 15 min post-orexin A administration. Furthermore, orexin-A administration resulted in significantly increased %MI compared with that in the NS group at 10, 15, and 20 min, and this effect occurred in a dose-dependent manner [10 min: *F*(3,20) = 5.379; 15 min: *F*(3,20) = 19.83; 20 min: *F*(3,20) = 5.049, *P* < 0.05–0.01, Figure 4]. Pre-injection of 5 μg of SB-334867 blocked the effect of 0.5 μg of orexin-A (*P* < 0.05–0.01, Figure 4). Injection of SB-334867 alone did not induce any changes in gastric motility (*P* > 0.05, compared with the NS group, Figure 4).

### Effect of Bilateral Subdiaphragmatic Vagotomy on Orexin-A Induced Changes in Gastric Contraction

**FIGURE 5:**
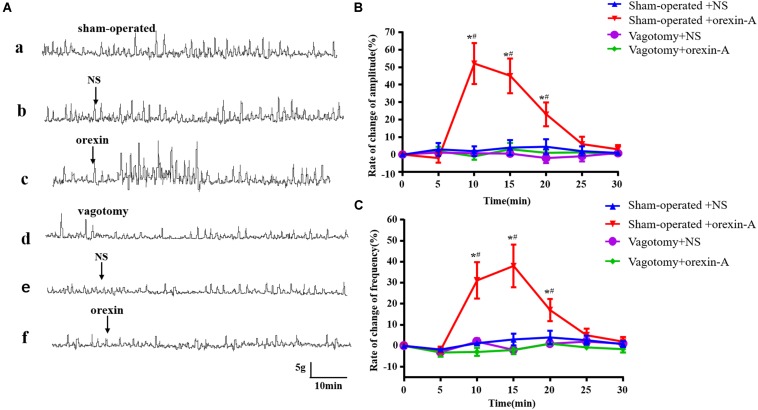
Effect of bilateral subdiaphragmatic vagotomy on orexin-A induced changes of gastric smooth muscle contraction. The represent recording of gastric motility in rats with sham operation and bilateral subdiaphragmatic vagotomy was shown in **(Aa,Ad)**, respectively. In the sham operation group, compared with NS group, orexin-A increased the amplitude and frequency of gastric contraction significantly **(Ab,c)**. However, this effect was abolished by bilateral subdiaphragmatic vagotomy **(Ae,f)**. Pooled data summarized change rate of amplitudes **(B)** and frequencies of the gastric contraction **(C)**. Percentage of the frequency and amplitude change was derived from the equation: frequency or amplitude change = (frequency or amplitude after microinjection - frequency or amplitude before microinjection)/frequency or amplitude before microinjection × 100%. Data are represented as mean ± SD, *n* = 6. Data were analyzed using two-way ANOVA followed by a Bonferroni *post hoc* test. *P < 0.01 vs. Sham-operated + NS group; ^#^*P* < 0.01 vs. Vagotomy + Orexin-A group. NS, normal saline.

Compared with the sham operation group, gastric contractility was significantly reduced following resection of the vagus nerve (Figures 5Aa,d). In the sham operation group, the frequency and amplitude of gastric contraction was significantly increased in response to 0.5 μg of orexin-A compared with those in the NS group [amplitude: 10 min: *F*(1,20) = 90.82; 15 min: *F*(1,20) = 87.65; 20 min: *F*(1,20) = 34.29; frequency: 10 min: *F*(1,20) = 45.45, 15 min: *F*(1,20) = 62.13, 20 min: *F*(1,20) = 26.58, *P* < 0.01, Figures 5Ab,c,B,C]. The latency of orexin-A was about 5–10 min, and the peak frequency of gastric contraction occurred at 15 min after administration. The peak amplitude occurred approximately 10 min after administration. In contrast, orexin-A did not significantly affect the frequency or amplitude of gastric contraction compared with those in the NS group following bilateral vagotomy (Figures 5Ae,f,B,C, *P* > 0.05). These results suggested that the effects of orexin-A in the CeA on gastric contraction were mediated by the vagus nerve.

### Effect of Orexin-A on Discharge of CeA GD Neurons

**FIGURE 6:**
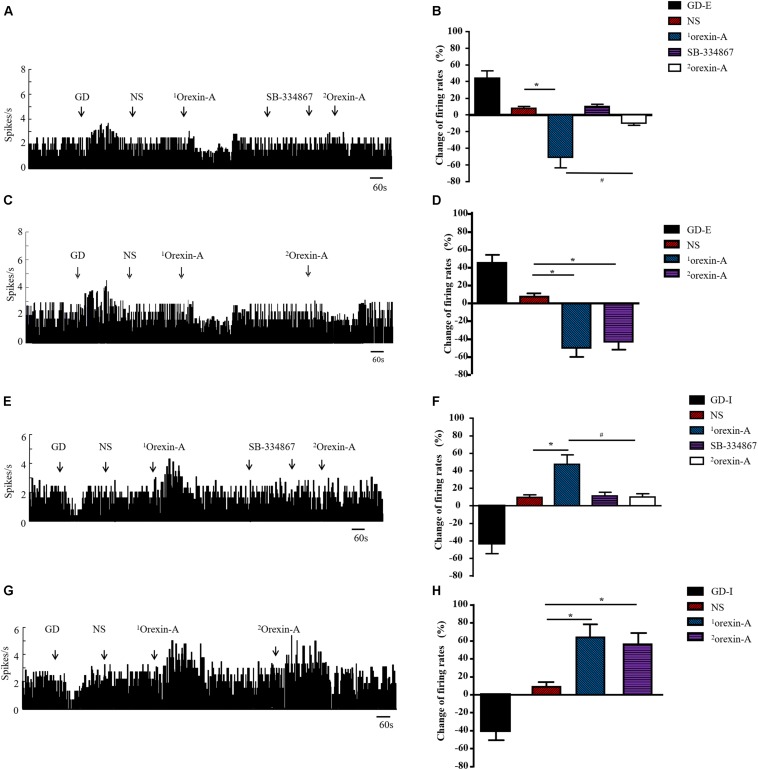
Effect of orexin-A on the discharge of GD neurons in CeA of rats. Typical frequency histograms showed that orexin-A changed discharge rate of GD-E **(A)** and GD-I **(E)** neurons in CeA. Pooled data summarized the effects of orexin on GD neurons in CeA **(B,F)**. After pretreatment with SB-334867, the orexin-A induced response was completely blocked and NS or SB-334867 alone had no effect on the firing frequency of GD neurons **(A,B,E,F)**. When orexin-A was given for the second time, it still had inhibitory or excitatory effect on GD-E and GD-I neurons respectively **(C,D,G,H)**. The effect of tested agents on neuronal discharge was calculated by (the maximal change of frequency within 50 s after treatment – average frequency 120 s before treatment)/(average frequency 120 s before treatment) × 100%. Data are shown as mean ± SD. Data were analyzed using Paired Student’s *t*-test to compare the difference of firing rate before and after treatment and Student’s *t*-test was used to compare the effect of two different treatment. *P < 0.05, compared with NS; ^#^*P* < 0.05, compared with ^1^orexin-A. GD, gastric distension; GD-E, gastric distension excitatory; GD-I, gastric distension inhibitory; NS, normal saline.

A total of 137 neurons were detected in 45 rats. Ninety-seven were GD responsive neurons, and 54 of these neurons exhibited increased firing frequency called as GD-E neurons. In contrast, 43 neurons were inhibited by GD, called as GD-I neurons. The remaining 40 neurons did not respond to GD. Orexin-A decreased firing frequency from 2.37 ± 0.69 Hz to 1.16 ± 0.27 Hz (*P* < 0.01) in 34 out of 54 (34/54, 62.97%) GD-E neurons and the average percent of decrease was 51.05 ± 12.21%, which was significantly different from that of the NS group (12.31 ± 6.67%, *P* < 0.01, Figures 6A,B). In addition, 7 (7/54, 12.96%) GD-E neurons were excited by orexin-A and 13 (13/54, 24.07%) neurons did not respond to orexin-A.

To determine whether the effects of the second administration of orexin-A were diminished due to desensitization of the orexin receptor or due to the effects of receptor blockers, we evaluated the effects of a second microinjection of orexin-A without SB-334867 pretreatment. Twelve GD-E neurons were recorded, and orexin-A treatment resulted in a discharge frequency decrease from 2.45 ± 0.67 Hz to 1.24 ± 0.31 Hz (*P* < 0.01, Figures 6C,D), and an average percent decrease of 49.39 ± 10.21%, which was significantly different than that in the NS group (7.71 ± 2.33%, *P* < 0.01). The second administration of orexin-A decreased the discharge frequency from 2.45 ± 0.67 Hz to 1.39 ± 0.37 Hz (*P* < 0.01, Figures 6C,D), and the average percent decrease was 43.27 ± 8.89%, which was not significantly different from that observed in response to the first administration of orexin-A (49.39 ± 10.21%, *P* > 0.05).

Of the 43 GD-I neurons observed in this study, orexin-A excited 29 (29/43, 67.44%) with a discharge frequency increase from 2.73 ± 0.76 Hz to 4.03 ± 1.34 Hz (*P* < 0.01). The average percent increase was 47.62 ± 10.41%, which was significantly different from that in the NS group (9.82 ± 2.86%, *P* < 0.01, Figures 6E,F). In addition, 6 (6/43, 13.95%) GD-I neurons were inhibited by orexin-A and 8 (8/43, 18.61%) GD-I neurons did not respond to orexin-A. In another group, thirteen GD-I neurons were recorded, and orexin-A induced an increase in discharge frequency from 2.63 ± 0.82 Hz to 4.31 ± 1.44 Hz (*P* < 0.01, Figures 6G,H), with an average percent increase of 63.88 ± 14.41%, which was significantly different from that in the NS group (8.48 ± 2.86%, *P* < 0.01). The second application of orexin-A resulted in an increase in discharge frequency from 2.63 ± 0.82 Hz to 4.11 ± 1.32 Hz (*P* < 0.01, Figures 6G,H) and the average percent increase was 56.27 ± 12.27%, which was not significantly different from that following the first administration of orexin-A (63.88 ± 14.41%, *P* > 0.05).

Pre-administration of 5 μg of SB-334867 completely inhibited the effects of 0.5 μg of orexin-A on GD neurons in the CeA, but SB-334867 alone did not induce changes in discharge of GD neurons (Figures 6A,B,E,F).

### Effect of Microinjection of Orexin-A Into the CeA on C-fos Expression

**FIGURE 7:**
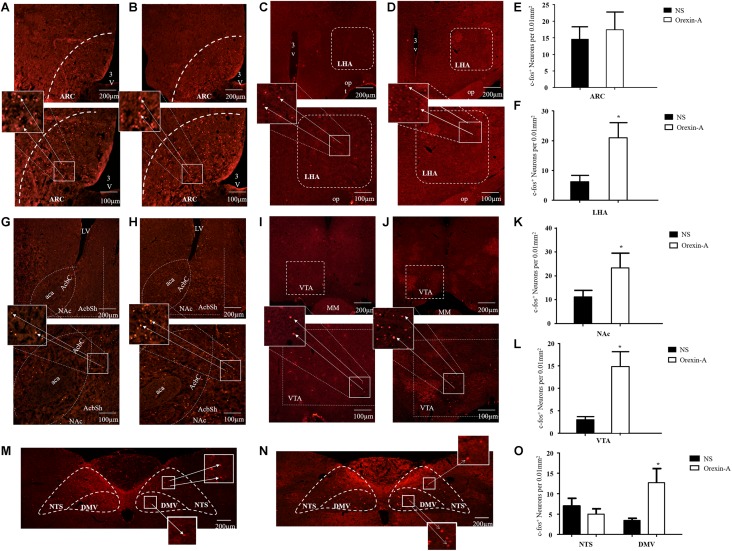
Effect of microinjection of orexin-A into CeA on expression of c-fos in ARC, LHA, NAc, VTA, NTS, and DMV. Representative expression of c-fos immunoreactive nuclei in ARC in control **(A)** and orexin-A **(B)** group, in LHA in control **(C)** and orexin-A **(D)** groups, in NAc in control **(G)** and orexin-A **(H)** groups, in VTA in control **(I)** and orexin-A **(J)** group, in NTS and DMV in the control **(M)** and orexin-A **(N)** group. Summary of expression of c-fos positive neurons was shown in **(E,F,K,L,O)** and the number of c-fos positive neurons was counted with a Macintosh-based image analysis system. Data are represented as mean ± SD, *n* = 8. Data were analyzed using Student’s *t*-test. *P < 0.05 vs. NS group. ARC, arcuate nucleus; LHA, lateral hypothalamic area; NAc, accumbens nucleus; VTA, ventral tegmental area; NTS, nucleus of the solitary tract; DMV, dorsal motor nucleus of the vagus; NS, normal saline.

To characterize the brain regions involved in the effects of orexin-A in the CeA, we evaluated the expression of c-fos protein in three feeding- and gastric motility-related brain areas including the hypothalamus, reward-related nuclei, and the DVC. The results showed that the c-fos protein mainly expressed in the LHA, the ARC, the VTA, the NAc, the DMV, and the NTS. The expression levels of c-fos in the LHA (orexin-A group: 21.02 ± 5.11 cells/0.01 mm^2^, NS group: 6.22 ± 2.12, *P* < 0.05, Figures 7C,D,F), the NAc (orexin-A group: 23.45 ± 6.11 cells/0.01 mm^2^, NS group: 11.37 ± 3.12 cells/0.01 mm^2^, *P* < 0.05, Figures 7G,H,K), the VTA (orexin-A group: 15.11 ± 3.33 cells/0.01 mm^2^, NS group: 3.57 ± 0.78 cells/0.01 mm^2^, *P* < 0.05, Figures 7I,J,L), and the DMV (orexin-A group: 13.27 ± 3.45 cells/0.01 mm^2^, NS group: 3.56 ± 0.56 cells/0.01 mm^2^, *P* < 0.05, Figures 7M–O) were significantly increased following microinjection of orexin-A into the CeA. However, in the ARC (orexin-A group: 17.52 ± 5.33 cells/0.01 mm^2^, NS group: 14.53 ± 4.23 cells/0.01 mm^2^, *P* > 0.05, Figures 7A,B,E) and the NTS (orexin-A group: 5.23 ± 1.33 cells/0.01 mm^2^, NS group: 7.15 ± 1.92 cells/0.01 mm^2^, *P* > 0.05, Figures 7M–O), orexin-A did not induce any significant changes in the expression of c-fos compared with that in the control group.

## Discussion

The present study showed that microinjection of 0.5 or 5 μg of orexin-A into the CeA did not significantly affect chow food intake, but significantly increased palatable high fat food intake. This effect was partially mediated by the dopamine D1 receptor. Microinjection of orexin-A into the CeA also accelerated gastric emptying and gastric motility in rats. These effects may have been related to regulation of the activity of gastric-related neurons in the CeA, and vagotomy abolished the effect of orexin-A on gastric motility. Furthermore, c-fos expression increased in the VTA, the NAc, the LHA, and the DMV, which suggested that these nuclei might play role in orexin-A-mediated effects in the CeA directly or indirectly.

Recent studies have shown that the CeA modulates appetitive behaviors (Cai et al., 2014; Kim et al., 2017; Fadok et al., 2018). Identification of neuronal subpopulations in the CeA that regulate food intake still remains elusive. Pang et al. (2015) reported that secretin suppressed food intake through modulation of spontaneous firing of neurons in the CeA. Another study showed that pituitary adenylate cyclase-activating polypeptide (PACAP) in the CeA inhibited food intake through melanocortin and the TrkB pathway (Iemolo et al., 2015). In contrast, chronic stress-induced obesity has been shown to be closely associated with activation of NPY neurons in the CeA, which resulted in enhanced food intake and reduced energy expenditure (Ip et al., 2019). Furthermore, CeA opioid receptors have been shown to mediate consumption of a palatable high-fat diet in fasting rats (Parker et al., 2014). Therefore, the different subtypes of neurons in the CeA play different roles in feeding regulation, and different metabolic states may result in different CeA functions. In the present study, we found that exogenous orexin-A injected into the CeA did not affect chow food intake, but significantly increased palatable HFF intake in rats. Although the CeA is involved in the feeding and reward systems (Douglass et al., 2017; Tom et al., 2019), our results demonstrated that orexin-A in the CeA regulated hedonic feeding rather than homeostatic feeding. Similar to our findings, Dube et al. (1999) reported that microinjection of orexin-A into the CeA failed to stimulate feeding in SD rats. In contrast, another study reported that infusion orexin-A into the CeA markedly increased food intake in hamsters (Alo et al., 2015). This discrepancy may have resulted from species differences and different recording timing. Many studies have shown that the orexinergic system is involved in regulation of hedonic feeding. For example, acute high fat diet consumption activated the mesolimbic pathway, and orexinergic neurons in the LHA participated in this reward-related process via OX1R (Valdivia et al., 2014). Furthermore, a recent study reported that prepronociceptin-expressing neurons in the CeA promoted palatable HFF consumption and reward seeking through connections with the parabrachial nucleus (PBN), the ventral bed nucleus of the stria terminalis (BST), and the nucleus of the solitary tract (NTS) (Hardaway et al., 2019). In the present study, we also showed that microinjection of orexin-A into the CeA enhanced palatable HFF intake in rats. In addition, we showed that this effect was blocked by the OX1R antagonist SB-334867, which suggested the orexin-A-enhanced palatable HFF intake was mediated by OX1R. Many studies have shown that hedonic feeding is regulated by the reward system, and the VTA, the NAc, and the PFC are key components in this system. Dopamine neurons in the VTA project to the limbic forebrain, including the amygdala, the NAc, the hippocampus, as well as the PFC, and it is the predominant neuronal substrate of the limbic system responsible for reward, motivation, and addiction (Nestler and Carlezon, 2006; Luscher and Malenka, 2011). The dopamine D1 receptor antagonist SCH-23390 attenuated the effects of orexin-A in our study, which suggested the dopaminergic system participated in this process. Furthermore, our immunofluorescence staining results showed that microinjection of orexin-A into the CeA increased c-fos expression in the VTA and the NAc. So, it is plausible that orexin-A acted on neurons in the CeA through OX1R and this signal then propagated to the VTA and the NAc. Thus, the activation of the reward system enhanced the consumption of palatable HFF. However, innervation of the VTA by the CeA is minimal (Zahm et al., 1999), and no studies have shown that the CeA has any direct connections with the NAc. In this context, increased c-fos expression in the VTA and the NAc in response to microinjection of orexin-A in the CeA was likely due to indirect signaling. These indirect pathways require further characterization. In addition, identification of the types of OX1R^–^expressing neurons in the CeA involved in palatable HFF intake, and the types of neurons in the VTA and the NAc activated by OX1R-expressing neurons in the CeA is necessary to aid in understanding of the mechanisms of reward-driven food intake and prevention of obesity.

The LHA is one of the most important brain areas that participate in regulating food intake. Studies have shown that intra-LHA injection of neurotransmitter analogs or their receptor antagonists could induce feeding or suppress feeding (Stuber and Wise, 2016). Furthermore, LHA neurons send GABAergic and glutamatergic fibers to the VTA to modulate the activity of neurons in VTA suggests that neurons in the LHA may be involved in hedonic feeding. Morphological study shows that the CeA projects directly to the dorsal region of the LHA (Reppucci and Petrovich, 2016). Therefore, increased c-fos expression in the LHA might have occurred directly in response to the effects of orexin-A in the CeA, which suggested that CeA OX1R receptor-positive neurons may participate in rewarding food intake via neurons in the LHA.

Our studies, and those by other groups, have shown that central administration of orexin-A resulted in increased gastrointestinal motility and promoted gastric acid secretion in both physiological and pathophysiological states. For example, LHA orexin-A neurons enhanced gastric motility and gastric acid secretion through projections to PVN Y1 receptor-expressing neurons (Wang et al., 2019). Exogenous orexin-A injected into the ARC alleviated cisplatin-induced nausea and vomiting and promoted gastric motility in cisplatin-treated rats. This process required activation of the downstream NPY pathway (Guo et al., 2018). Nozu et al. (2012) reported that intracisternal injection of orexin-A significantly stimulated gastric motility in freely moving conscious rats, and this effect was mediated by the vagus nerve. Nozu et al. (2011) also found that central administration of orexin-A enhanced fecal pellet output and accelerate colonic motility in freely moving rats. In addition to the hypothalamus, emotion centers also play in important role in regulation of gastric motility, and bidirectional projections have been identified between the hypothalamus and the amygdala (Barbier et al., 2017, 2018; Yamamoto et al., 2018). In the present study, we found that microinjection of orexin-A into the CeA accelerated gastric emptying and gastric constriction in a similar manner to that of orexin-A injection into the hypothalamus (Luan et al., 2017; Guo et al., 2019). Xu et al. (2011) reported that motilin activated GD neurons in the ARC and enhanced gastric motility in concert with other feeding-related brain nuclei such as the hypothalamus paraventricular nucleus (PVN). Gong et al. (2014) found that ghrelin modulated activity of GD neurons in the lateral septum and contributed to regulation of gastric motility. In the present study, we found that GD neurons also existed in the CeA. Exogenous orexin-A microinjected into the CeA strengthened discharge of GD-I neurons and weakened discharge of GD-E neurons, and these contrasting effects may have coordinated with each other to enhance gastric motility. Though no direct evidence shows a relationship between the activity of GD-E or GD-I neurons and gastric motility, increased stomach tonic pressure exerted by gastric distension has been shown to increase gastric emptying and gastric contraction (Strunz and Grossman, 1978; Lu et al., 2010). Therefore, as GD neurons receive mechanical signals induced by gastric distension, GD neurons may be involved in regulation of gastric motility, and orexin-A may enhance gastric motility via GD neurons. Our observation that the OX1R antagonist SB-334867 blocked the effects of orexin-A suggested orexin-A signaled through OX1R.

Brainstem vagovagal parasympathetic neural circuits play an important role in CNS-mediated control of stomach motility (Lu et al., 2019). Vagovagal neural circuits include the NTS, the DMV, and the area postrema (AP). Neurons in the NTS receive visceral sensory information from the gastrointestinal tract, and integrate this information with information from higher CNS centers including the limbic system and the hypothalamus to modulate regulation of physiological function (Grill and Hayes, 2012; Cui et al., 2018). The DMV is the output of the DVC. Efferent fibers from the DMV innervate postganglionic neurons located in the stomach and intestines, allowing for modulation of gastrointestinal motility and function. There are anatomical and physiologic evidences show that CeA is connected to the DVC, including NTS and DMV (Liubashina et al., 2000; Zhang et al., 2003; Cassidy et al., 2019). So, increased c-fos expression in DMV might have occurred directly in response to the effects of orexin-A in the CeA. We also evaluated the effects of orexin-A injected into the CeA on gastric motility in rats subjected to subdiaphragmatic vagotomy. The results showed that vagotomy abolished the effects of orexin-A, which supported our hypothesis that orexin-A injected into the CeA signaled through the DMV, and descending conduction was mediated by vagal nerves. Although the expression of c-fos protein did not change significantly in the NTS, it does not mean orexin-A in CeA had no effect on NTS for c-fos expression did not reflect inhibitory effects on neurons. Neurons in the CeA may connect to the NTS via inhibitory projections, which could suppress the activity of NTS GABA neurons. In turn, inhibitory effects in NTS resulted in excitation of neurons in the DMV.

In this study, we did not identify the types of cells that expressed c-fos, and alteration in c-fos expression in the brain regions may have been due to indirect effects that were not related to modulation of feeding and gastric motility. Future studies will characterize the types of neurons associated with the effects of orexin-A in the CeA, and the efferent projections from orexin receptor-expressing neurons in the CeA will be evaluated.

## Conclusion

The present study showed that microinjection of exogenous orexin-A into the CeA promoted HFF intake and enhanced gastric motility. These effects may have been related to the regulation of activity of GD-related neurons *via* OX1R. Furthermore, the effects of orexin-A on palatable HFF intake were partially mediated by the dopamine D1 receptor, and neurons in the LHA, the VTA, and the NAc may have been involved in this process directly or indirectly. The effect of orexin-A on gastric motility was mediated by the vagus nerve. Furthermore, the patterns of alterations in c-fos expression indicated that the CeA-DMV-vagus nerve–gastrointestinal tract axis might be critical to orexin-A-induced modulation of gastric motility.

## Data Availability Statement

The datasets generated for this study are available on request to the corresponding author.

## Ethics Statement

The animal study was reviewed and approved by Institutional Animal Care and Use Committee of Qingdao University.

## Author Contributions

XS was responsible for the conception, design, and revision of the article. TJ, FG, SG, and XL acquired the data. ZJ and ZQ undertook the statistical analysis and interpretation of the data. TJ wrote the first draft of the manuscript. LX helped to revise the manuscript. All authors contributed to and have approved the final manuscript.

## Conflict of Interest

The authors declare that the research was conducted in the absence of any commercial or financial relationships that could be construed as a potential conflict of interest. The reviewer YG declared a past co-authorship with one of the authors XS to the handling Editor. The reviewer YG declared a shared affiliation, with no collaboration with the authors to the handling Editor.
